# Feedback Valence Agency Moderates the Effect of Pre-service Teachers’ Growth Mindset on the Relation Between Revising and Performance

**DOI:** 10.3389/fpsyg.2019.01794

**Published:** 2019-08-13

**Authors:** Maria Cutumisu

**Affiliations:** Department of Educational Psychology, Centre for Research in Applied Measurement and Evaluation, University of Alberta, Edmonton, AB, Canada

**Keywords:** mindset, feedback agency, choice, learning, game

## Abstract

It is often assumed that having a choice in the learning process may benefit performance and learning. Concomitantly, it is believed that learning choices (e.g., seeking critical or confirmatory feedback) are due to mindset. However, the relation between choices and mindset is still a matter of debate: it is not known whether mindset interferes with the decision to seek critical feedback, the response to critical feedback, or both. This experiment investigates for the first time whether feedback valence agency moderates the effect of mindset on the relation between learning behaviors and learning outcomes. Participants were *n* = 120 pre-service teachers who were randomly assigned to one of two conditions, Choose (*n* = 68) and Assign (*n* = 52), and designed three posters in Posterlet, a game that assessed their learning behaviors (critical feedback and revising) and poster performance. Then, they completed a learning post-test that also included a mindset survey. Results reveal similar non-significant correlation patterns of mindset with learning behaviors and learning outcomes in both conditions. Feedback valence agency (i.e., condition) moderates the effect of growth mindset on the relation between revision and performance: students who choose to revise their posters more often (i.e., at least twice) perform significantly better when they endorse higher rather than lower levels of growth mindset but only when feedback valence is chosen rather than assigned. Theoretical implications indicate that feedback valence agency moderates the effect of growth mindset in driving how students *respond* to their own learning choices to improve their performance.

## Introduction

There is reason to believe that having some degree of control or agency over one’s learning yields positive learning behaviors and outcomes. According to Gallagher (2000), agency is defined as an individual’s sense of “causing or generating an action” (as cited in Arzy and Schacter, 2019). It is believed that development of agency over one’s learning is associated with increased self-regulation in learning contexts (Marulis et al., 2019), which is essential for lifelong learning. It is also assumed that attitudes toward constructive feedback, such as a learner’s mindset (i.e., the belief that intelligence or ability can be either stable or improved with effort; Dweck and Leggett, 1988; Dweck, 2017), influence learning outcomes. Moreover, it is thought that individuals’ responses to mistakes depend on their mindset (Moser et al., 2011). However, there is no evidence that feedback valence agency (i.e., control over receiving either confirmatory or critical feedback) after completing a task is related to mindset and whether, in turn, mindset shapes the relation between learning behaviors and learning outcomes. In psychology, valence refers to an intrinsic aversiveness or attractiveness of an event, object, or situation (Frijda, 1986). Here, critical feedback denotes negative (i.e., disconfirming, corrective) feedback, while confirmatory feedback denotes positive (i.e., reinforcing) feedback.

The contribution of each of the factors such as agency and mindset to learning outcomes is not clearly differentiated in the literature, although there is some evidence that they each may enhance learning independently. In general, there is a paucity of research examining the *relations* among feedback valence agency, mindset, and feedback-seeking choices (i.e., choosing between critical and confirmatory feedback). Research suggests that critical feedback is not always beneficial for performance (Kluger and DeNisi, 1996, 1998; Hattie and Timperley, 2007). This could be due to factors such as mindset, self-worth, ego-threat, or stereotype threat (Rydell and Boucher, 2017; Zingoni and Byron, 2017). For instance, it was found that threats to self-worth hindered individuals’ engagement with critical feedback (Critcher et al., 2010). The overarching goal of this research is to identify factors that support students in achieving better performance and learning outcomes when they have a choice over their feedback valence and when they are assigned their feedback valence, respectively. Hence, this experimental study was designed to elucidate whether individuals’ choices over their feedback valence influence the effect that mindset exerts on the relation between learning behaviors and learning outcomes. Does mindset moderate the relation between learning behaviors and learning outcomes differentially when students choose or are assigned critical or confirmatory feedback?

In previous research, convergent validity of learning choices was demonstrated (i.e., critical feedback-seeking and revision choices correlated with several internal and external learning outcomes; Cutumisu et al., 2019). In a similar study where all students had a choice regarding their feedback valence, discriminant validity (Campbell and Fiske, 1959) of learning choices was established by revealing that critical feedback-seeking shows a different pattern of correlations with the choice to revise and with performance than mindset (Cutumisu, 2019), which is generally believed to be another relevant predictor of performance. Specifically, although critical feedback-seeking was associated with both performance and choosing to revise, mindset was not associated with performance or the choice to revise. However, the prior study design did not enable the drawing of a causal relation between feedback valence agency and the effect of mindset on the relation between learning behaviors and learning outcomes. The present research extends the previous study by proposing an experimental design in which the agency of students’ feedback valence is manipulated. It investigates whether mindset and learning behaviors (i.e., seeking or being assigned critical feedback as well as choosing to revise) show a different pattern of correlations with learning outcomes (i.e., both performance and learning measures are employed in the current study, whereas only performance was used in the previous study) in two situations: when students can choose their feedback valence and when the feedback valence is assigned to them. The experiment examines if feedback valence agency (i.e., whether students choose or are assigned critical feedback) moderates the effect of mindset (growth and fixed, respectively) on the relations between students’ learning behaviors and learning outcomes, hypothesizing that agency matters in how individuals’ growth mindset moderates the relation between their learning behaviors and learning outcomes. Specifically, the experiment was designed to answer the following research questions:

1. Is mindset associated with learning behaviors and learning outcomes in each condition?
2. Does condition moderate the effect of mindset on the relation between learning choices?
3. Does condition moderate the effect of mindset on the relation between learning choices and learning outcomes?

The rest of the manuscript is organized as follows. First, the conceptual background underlying this research is outlined, including a brief description of Posterlet, a game designed to collect participants’ feedback-seeking and revising choices, followed by a description of the mindset post-test. Second, an empirical experiment is presented that explores the moderating role of feedback valence agency in shaping the effect of mindset over the relation between learning behaviors and learning outcomes for pre-service teachers (i.e., undergraduate students who aspire to become teachers by pursuing an education program). Finally, the results of the study are presented and discussed, followed by theoretical and practical implications.

## Conceptual Background

Mindset is a motivational construct believed to be central to individuals’ meaning-making system, especially when they attempt to make sense of their learning. Dweck’s (1999) mindset theory distinguishes two beliefs or implicit theories that individuals hold regarding human qualities or attributes (Dweck and Leggett, 1988), such as intelligence or ability (e.g., mathematics; Good et al., 2012). Specifically, fixed mindset is an entity belief that individuals’ attributes are fixed (i.e., individuals are not capable of change), while growth mindset is an incremental belief that individuals’ attributes are malleable and they can be improved with effort (i.e., individuals are capable of change; Dweck and Leggett, 1988; Dweck, 1999, 2017; Martin, 2015).

These beliefs are thought to influence individuals’ propensity for self-directed learning, their desire to pursue difficult challenges (Lee et al., 2012), as well as their responses to challenges by orienting them toward certain activities that highlight their personal characteristics. For instance, it is thought that growth mindset promotes more openness toward engaging with critical feedback, likely because individuals perceive critical feedback as an agent of growth and as a step in improving their performance. Individuals who are comfortable with critical feedback are better positioned to understand their own abilities and to correct their mistakes. They also tend to attribute failure to factors within their control, such as effort (Dweck, 1999, 2006). Thus, they tend to embrace failure and tend to be more mastery-oriented (i.e., motivated to develop competence) than performance-oriented (i.e., motivated to validate competence) by actively seeking challenges and focusing on the learning process (Lou, 2019). Additionally, learners who perceive performance as mutable, rather than due to inherent ability, may be more likely to use feedback to improve their performance. Furthermore, they may be more likely to change their characteristics when their environment presents them with motivation, opportunity, and instruction (Carr et al., 2012) by working hard and persevering. More recently, growth mindset was found to predict adaptive learning behaviors (Yeager et al., 2011, 2016a; Yeager and Dweck, 2012; Claro et al., 2016).

In contrast, fixed mindset promotes a preference for confirmatory feedback (Ehrlinger et al., 2016), likely to avoid failure and protect one’s self-esteem. Fixed mindset may determine individuals to avoid situations in which they can receive critical feedback, and it also promotes a diminished attention to critical feedback (Mangels et al., 2006; Williams and Ehrlinger, 2017) that is viewed as being unpleasant to receive and may not necessarily provide immediate benefits (Freitas et al., 2004). It can also determine individuals to avoid expanding effort on tasks as a way to justify failure (Nussbaum and Dweck, 2008). As they view their capabilities as immutable, individuals who endorse a fixed mindset could also be less likely to revise their work when they are provided with constructive feedback. They also tend to attribute failure to factors outside their control (e.g., luck) and to their own lack of innate aptitude, displaying diminished willingness to confront bias (Carr et al., 2012) and to seek challenging situations that may reveal incompetence, when competence is perceived as low (Hong et al., 1999). Finally, they tend to be more performance-oriented, being interested in outperforming others rather than improving their own learning.

Although mindset could be related to students’ learning behaviors, it is not clear whether mindset relates to students’ performance. Researchers recommend a more in-depth exploration of this relation (Damgaard and Nielsen, 2018), especially as recent meta-analyses found a weak relation between mindset and performance (Sisk et al., 2018).

*Self-Determination Theory* (SDT; Ryan and Deci, 2000) is a motivational theory that serves to inform this study, as the question at the core of this experiment explores whether individuals’ sense of agency toward the valence of feedback plays a role in the moderating effect of mindset on the relation between learning behaviors and learning outcomes. It posits that motivation, self-regulation, and well-being depend on an individual’s innate psychological needs of competence, autonomy, and relatedness. Relevant to the current research, choices and self-direction in learning enhance intrinsic motivation through perceived autonomy (Deci and Ryan, 1985). Thus, the opportunity to choose one’s feedback valence seems better positioned to motivate individuals to engage with feedback more meaningfully and be willing to revise and improve their work.

### Posterlet

We employed two versions of the Posterlet game (Cutumisu et al., 2015) in this experimental study; the yoked design of the present experiment was described in detail in prior research (Cutumisu and Schwartz, 2014). Although students’ task in Posterlet is to design posters, the game does not explicitly teach graphic design principles. Instead, students learn about graphic design principles from the feedback they encounter in the game on each of their posters and they have total freedom in terms of their poster designs.

**Figure 1:**
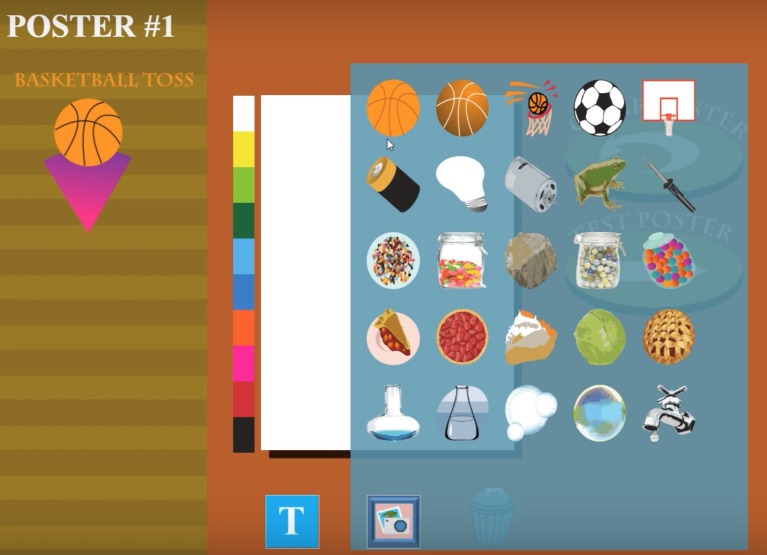
In Posterlet, players can design posters using pre-determined text and images.

The game starts with a sequence of steps displayed individually that provide the back story: students need to create digital posters that will be posted at three booths to ascertain if they garner interest from the Fall Fun Fair participants (i.e., sell tickets). Each time, students choose a booth theme (out of five possible themes) and design a poster for it, choosing pre-determined images and text from two widgets placed at the bottom of the poster canvas, as shown in Figure 1.

**Figure 2:**
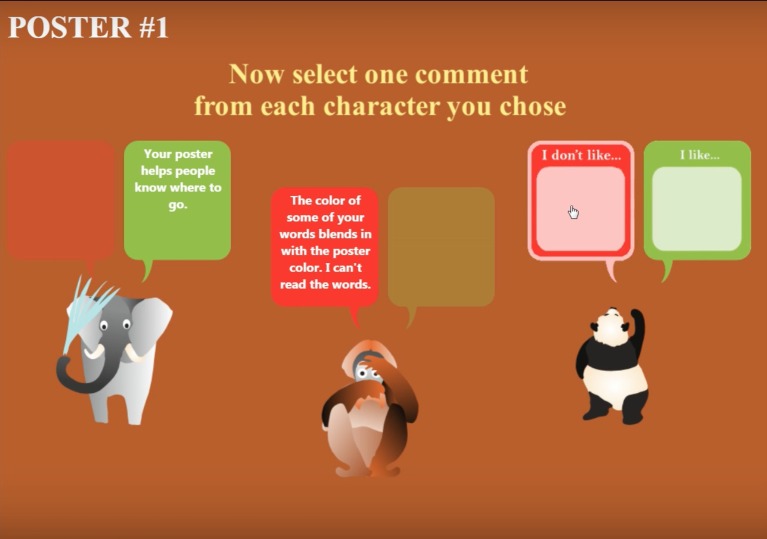
In the Choose condition, players choose either critical or confirmatory feedback from each character.

Then, they select three virtual characters from a focus group. Following that, in the Choose condition, players choose either critical feedback (“Where is the Fall Fair going to be?”) or confirmatory feedback (“Your poster helps people know where to go.”) from each character about their poster, as shown in Figure 2.

**Figure 3:**
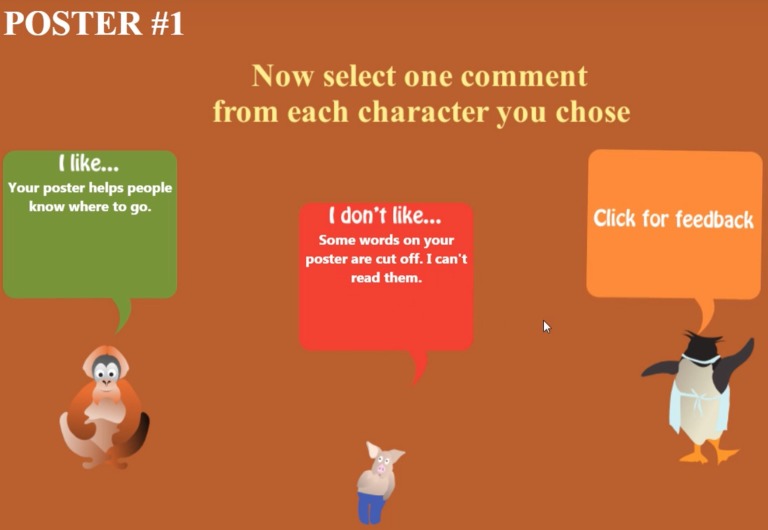
In the Assign condition, players click on the orange box (“Click for feedback”) to reveal the feedback assigned to them, through a yoked experimental design, of the same valence and order of a matched participant in the choose condition.

In the Assign condition, players are assigned the choices of participants in the Choose condition (i.e., either critical or confirmatory feedback about their poster), according to a yoked-design schedule, as shown in Figure 3.

The feedback generated is tailored to each student, as it depends on each student’s poster. The game analyzes each poster against a set of 21 graphic design rules (e.g., the location of the fair is included, the text is large enough, etc.). It awards a point for every rule used correctly, it subtracts a point for every rule used incorrectly, and it assigns zero points for any rule that is not used on the poster (e.g., if the poster does not include images, all the rules concerning images will be assigned zero points). Then, it sums the scores of each rule, generating a poster quality score that ranges from −21 to 21 for each poster. The game’s feedback system is described in detail in prior research (Cutumisu et al., 2017). Then, students choose either to revise their poster or to submit it unchanged. Finally, the number of tickets sold (i.e., poster performance) is displayed. In addition to measuring students’ cognitive outcomes (i.e., learning performance of graphic design principles) by computing an overall poster performance score, Posterlet’s main goal is to track non-cognitive outcomes: the amount of critical feedback that students encounter as well as the number of times they choose to revise their posters.

## Materials and Methods

### Participants, Procedure, and Data Sources

Table 1 comprises the study and participant information for the *n* = 120 pre-service students included in this study. After informed consent was secured, participants were assigned randomly to either the Choose or the Assign condition.

**Table 1:** Participant and study information.

Condition	Gender		Total	Age range in years	*M*_age_ (SD) in Years	*M*_Design Duration_ (SD) in minutes
	Female	Male				
Choose	39	29	68	20–41	24.47 (5.06)	11.10 (5.45)
Assign	29	23	52	19–50	25.63 (6.14)	10.45 (4.10)
Total	68	52	120	19–50	24.98 (5.56)	10.81 (4.89)

In each condition, students played a version of the three-round digital assessment game in which they designed posters and interacted with virtual characters in the game to learn how they performed on their posters *via* feedback highlighting relevant principles of graphic design. In each game version, the player first chooses three animal game characters. In the Choose condition, the student could choose to hear confirmatory or critical feedback from each selected game character. In the Assign condition, players were assigned the same choices of participants in the Choose condition. The game collected learning analytics about students’ behaviors (e.g., number of critical feedback messages encountered, number of times students chose to revise their posters, etc.) and poster performance. Then, students filled a post-test that also included a four-item mindset survey tailored for the domain of poster design (Cutumisu, 2019). Some of the students did not complete the mindset survey questions and the log files of three of the students were corrupt. On a scale of 1 (strongly disagree) to 5 (strongly agree), four questions were probing students’ fixed mindset (“You cannot really change your abilities to design posters.” and “You can learn new things, but you cannot really change your ability to design posters”) and growth mindset (“You can always change your abilities to design posters” and “You can get better at designing posters with practice”).

### Measures

#### In-Game Learning Behaviors

*Critical Feedback* is the measure of the critical feedback (0–9) encountered in the game (either by choosing it or by being assigned). *Revision* is the measure of the number of posters revised (0–3) in the game.

#### In-Game Performance

*Poster Quality* is the measure of performance in the game. Each of the three posters is evaluated according to graphic design rules and one point is awarded for a correct application of the rule, while one point is subtracted for an incorrect application of that rule on a poster. *Pre-test* measures the quality of the first poster, before revision.

#### In-Game Learning

*Poster Ranking* measures students’ learning of graphic design principles, as measured by an independent post-test (i.e., separate from the Posterlet game) described in prior research (Cutumisu and Schwartz, 2014; Cutumisu et al., 2019). This measure was introduced to capture students’ learning, not only poster performance as in prior research (Cutumisu, 2019), as a way to offer a more in-depth view of learning outcomes, the performance being part of the assessment environment (i.e., Posterlet), while the post-test being independent of the assessment environment. The distinction between performance and learning has been highlighted in the related literature (Soderstrom and Bjork, 2015). The post-test comprised four sets of poster pairs (a poster and a variation of that poster) and measures students’ ability to distinguish between correctly-used versus incorrectly-used graphic design principles. Students compared the posters in each pair to each other and decided which one they perceived as being the best. One of the two posters contained a mistake (a graphic design principle was used incorrectly, such as displaying a low contrast between the colors of the text and of the poster background). Each correct response was marked with 1 and each incorrect response with 0. Thus, this measure ranges from 0 to 4.

#### Mindset

*Growth Mindset* measures the belief that intelligence can be improved with learning and effort and it sums the two growth mindset items. In the Choose condition, the reliability of the two growth-mindset variable scale was high: rho = 0.66, *p* < 0.001, *α* = 0.79, *n* = 66. In the Assign condition, the reliability of the two growth-mindset variable scale was satisfactory: rho = 0.52, *p* < 0.001, *α* = 0.57, *n* = 52. *Fixed Mindset* measures the belief that intelligence is a stable entity (Dweck, 1999) and it sums the two fixed mindset items, as described in previous research. In the Choose condition, the reliability of the two fixed-mindset variable scale was high: rho = 0.73, *p* < 0.001, *α* = 0.84, *n* = 66. Also, in the Assign condition, the reliability of the two fixed-mindset variable scale was high: rho = 0.78, *p* < 0.001, *α* = 0.72, *n* = 52. The decision to consider two different mindset constructs rather than a composite mindset construct was discussed in prior research and it is still an open research question in the mindset literature (Cutumisu, 2019).

## Results

### Is Mindset Associated With Learning Behaviors and Learning Outcomes in Each Condition?

Tables 2, 3 include the Spearman correlations of the non-normally distributed mindset variables with in-game learning behavior measures and learning outcome measures (i.e., poster performance and learning of graphic design principles), conducted per condition. There were no significant associations among our variables across conditions.

**Table 2:** Spearman correlations between mindset and learning measures (Choose^*^ condition).

Measures (*n* = 63)	Critical feedback	Revision	Poster quality	Poster ranking
Growth mindset	0.15	0.07	−0.03	0.04
Fixed mindset	−0.01	−0.04	−0.03	−0.09

* *Except for Poster Ranking, results reported in Cutumisu (2019) are included here for convenience*.

**Table 3:** Spearman correlations between mindset and learning measures (Assign condition).

Measures (*n* = 52)	Critical feedback	Revision	Poster quality	Poster ranking
Growth mindset	−0.06	0.12	0.12	0.02
Fixed mindset	−0.02	−0.17	−0.02	0.09

### Does Condition Moderate the Effect of Mindset on the Relation Between Learning Choices?

Three-way analyses of variance (ANOVAs) were conducted to determine whether the experimental condition moderated the effect of mindset (growth and fixed, respectively) on the relation between the two choices (Critical Feedback and Revision). Analyses were repeated using the Sidak and Bonferroni corrections for simple main-effects tests, respectively.

Mindset variables (fixed and growth) and behaviors (critical feedback and revision) were divided into higher and lower levels, based on a median split. The lower level of critical feedback ranged from 0 to 5, while the higher level of critical feedback ranged from 6 to 9, as in prior research (Cutumisu, 2019). The latter range is equivalent with encountering critical feedback more often than confirmatory feedback across the game. The lower level of revision ranged from 0 to 1, while the higher level of revision ranged from 2 to 3. The lower level of growth mindset ranged from 2 to 8, while the higher level of growth mindset ranged from 9 to 10. Finally, the lower level of fixed mindset ranged from 2 to 3, while the higher level of fixed mindset ranged from 4 to 10, consistent with prior research (Cutumisu, 2019).

Condition (i.e., feedback valence agency), critical feedback group, and growth mindset group were used as categorical independent variables to predict the dependent variable, Revision. Results revealed no main effects and no two-way interactions. There was no significant three-way interaction of condition, growth mindset group, and critical feedback group: *F*(1, 107) = 3.00, *p* = 0.09, $\eta_{p}^{2}$ = 0.03 in predicting Revision. A similar three-way analysis for fixed mindset revealed no main effects of condition, fixed mindset group, critical feedback group, and no two-way interactions. Similarly, there was no significant three-way interaction among these three variables predicting Revision: *F*(1, 107) = 1.96, *p* = 0.16, $\eta_{p}^{2}$ = 0.02.

### Does Condition Moderate the Effect of Mindset on the Relation Between Learning Choices and Learning Outcomes?

Three-way analyses of variance and covariance, respectively, examined the effect of condition, mindset group (growth or fixed), and behavior (critical feedback group or revision group) on learning outcomes (poster performance, controlling for the pre-test, and learning of graphical design principles).

#### Feedback Valence Agency, Growth Mindset, Critical Feedback, and Learning Outcomes

Two types of analyses, a three-way analysis of covariance (ANCOVA) and a three-way analysis of variance (ANOVA), respectively, were conducted on the dataset containing the combined conditions (Choose and Assign) to examine the effect of condition, growth mindset group, and critical feedback group on poster performance, controlling for the pre-test, and on the learning of graphic design principles, respectively. Results of the ANCOVA revealed no main effects and no two-way interactions. Also, there was no significant interaction for Poster Quality: *F*(1, 106) = 2.40, *p* = 0.12, $\eta_{p}^{2}$ = 0.02. There were also no main effects, no significant two-way interactions, and there was no significant three-way interaction for Poster Ranking: *F*(1, 107) = 0.90, *p* = 0.35, $\eta_{p}^{2}$ = 0.01.

#### Feedback Valence Agency, Growth Mindset, Revision, and Learning Outcomes

A three-way analysis of covariance and a three-way analysis of variance were conducted on the entire dataset with the conditions combined to examine the effect of condition, growth mindset group (lower versus higher levels), and revision group (students who chose to revise at most one poster versus students who chose to revise more than one poster) on poster performance, controlling for the pre-test, and on the learning of graphic design principles, respectively. Results of the ANCOVA revealed a main effect for pre-test and a three-way interaction for Poster Quality: *F*(1, 106) = 9.22, *p* < 0.01, $\eta_{p}^{2}$ = 0.08. There were no other significant main effects or two-way interactions. Also, there were no main effects, no significant two-way interactions, and no significant three-way interaction for Poster Ranking: *F*(1, 107) = 1.63, *p* = 0.20, $\eta_{p}^{2}$ = 0.01.

Two-way analyses of covariance were conducted by condition to further explore the overall moderation result. In the Assign condition, there was a main effect of the pre-test but no interaction between Growth Mindset Group and Revision Group predicting Poster Quality: *F*(1, 47) = 2.90, *p* = 0.09, $\eta_{p}^{2}$ = 0.06. This contrasts the Choose condition results reported in prior research (Cutumisu, 2019), which revealed an interaction between Growth Mindset Group and Revision Group predicting performance, controlling for the pre-test. In the present research, to gain a better understanding of the significant result found previously in the Choose condition, simple main-effects analyses were conducted for this condition. Results indicate that Choose condition students who choose to revise their posters more often (i.e., more than once) across the game perform significantly better on their posters than those who revise their posters less often (i.e., at most once) when they endorse higher rather than lower levels of growth mindset. Also, Choose condition students who endorse higher levels of growth mindset perform significantly better on their posters than those who endorse lower levels of growth mindset when they choose to revise their posters more often (i.e., more than once) rather than less often (i.e., at most once) across the game.

Then, a two-way analysis of covariance was conducted per revision group. Results revealed a significant interaction between condition and growth mindset group predicting performance only for the lower revision group (i.e., 0–1): *F*(1, 46) = 6.92, *p* < 0.05, $\eta_{p}^{2}$ = 0.13. However, simple main-effects analyses revealed a marginal effect of condition for the students who endorsed a lower growth mindset and chose to revise less often, indicating that those who choose their feedback valence tend to outperform those who are assigned their feedback valence. Finally, simple main-effects analyses revealed that students who revised their posters more often (i.e., at least twice) and endorsed a higher growth mindset (i.e., 9–10) performed significantly better on their posters when they chose compared to when they were assigned their feedback valence.

#### Feedback Valence Agency, Fixed Mindset, Critical Feedback, and Learning Outcomes

Several three-way analyses of covariance and variance, respectively, used the independent categorical variables: Condition, Fixed Mindset Group (lower versus higher levels), and Critical Feedback Group (0–5 versus 6–9 critical feedback messages) to predict the dependent variable, Poster Quality, controlling for the pre-test, and learning of graphic design principles, respectively. Results of the ANCOVA revealed a main effect for pre-test but no two-way interactions. Also, there was no significant three-way interaction of condition, fixed mindset group, and critical feedback group predicting Poster Quality, controlling for the pre-tests: *F*(1, 106) = 1.06, *p* = 0.31, $\eta_{p}^{2}$ = 0.01. Results also revealed no three-way interaction of condition, fixed mindset group, and critical feedback group predicting Poster Ranking: *F*(1, 107) = 0.29, *p* = 0.59, $\eta_{p}^{2}$ = 0.003.

#### Feedback Valence Agency, Fixed Mindset, Revision, and Learning Outcomes

Several three-way ANCOVA and ANOVA analyses, respectively, using condition, fixed mindset group, and revision group to predict performance controlling for the pre-test and learning of graphic design principles were conducted. Results of the ANCOVA revealed no main effects or interactions. There was no interaction of condition, revision group, and fixed mindset group to predict performance controlling for the pre-test: *F*(1, 106) = 2.58, *p* = 0.11, $\eta_{p}^{2}$ = 0.02. There was also no interaction of condition, revision group, and fixed mindset group to predict learning of graphic design principles: *F*(1, 107) = 1.74, *p* = 0.19, $\eta_{p}^{2}$ = 0.02.

## Discussion, Limitations, and Future Research

This experiment examined the associations of mindset with learning behaviors and learning outcomes, controlling the way students interact with critical feedback (i.e., whether they choose it or it is assigned to them). The initial hypothesis was partly fulfilled, as results showed that feedback valence agency moderated the effect of growth mindset on the relation between one of the learning behaviors (revision) and one of the learning outcomes (performance) included in this experiment. This is aligned with the findings discussed in the literature review section showing that individuals respond to events differentially, according to the mindsets they endorse regarding a particular domain, and that individuals who endorse a growth mindset tend to invest more effort into their learning processes and outcomes.

### Feedback Valence Agency, Mindset, and the Relation Between the Two Learning Behaviors

In support to previous research that only focused on the Choose condition (Cutumisu, 2019) and revealed that mindset (both fixed and growth) does not influence students’ learning behaviors (i.e., decisions to seek critical feedback and to revise their posters), the current experiment confirms that mindset does not exert a direct influence over the choice to revise posters, regardless of whether students choose or are assigned their feedback valence. Indeed, results of the correlation analyses show a consistent pattern of results (i.e., no correlations among any of the measures) between mindset and the two learning behaviors across conditions. Thus, the correlation result for the Choose condition contradicts the commonly held belief that fixed mindset promotes a preference for confirmatory feedback (Ehrlinger et al., 2016), as students who had control over their feedback valence did not also endorse one type of mindset or the other. Please note that, in this experiment, critical and confirmatory feedback are inversely correlated, so the absence of associations between mindset and critical feedback is equivalent with the absence of associations between mindset and confirmatory feedback. Also, results indicate that mindset (growth or fixed) is not associated with decisions to revise one’s work, regardless of individuals’ feedback valence agency. The lack of correlation between fixed mindset and revising in both conditions is supported by studies showing that individuals endorsing a fixed mindset may give up in the presence of failure, represented here as the encounter with critical feedback (Blackwell et al., 2007). Thus, they likely may choose not to revise their posters as a result.

This result could be due to variables not currently measured (e.g., stress, test fatigue, confidence, self-esteem, etc.). Also, as students in the Assign condition do not have control over the valence of their feedback and as they may have different ability levels, it is possible that they may receive critical feedback more often even when they design good posters. In that case, Posterlet would generate uninformative feedback (e.g., “I do not like fairs”), which does not prompt poster revisions, as there is nothing to fix. Taken together, results imply that, once students encounter critical feedback (either by choosing it or by being assigned to them), mindset does not seem to influence directly their decision to revise their posters as a response to critical feedback, regardless of how students encountered that feedback.

### Feedback Valence Agency, Mindset, and the Relation Between Learning Behaviors and Outcomes

#### Feedback Valence Agency, Mindset, Critical Feedback, and Learning Outcomes

The experiment shows that condition does not moderate the effect of mindset (growth or fixed) on the relation between critical feedback and learning outcomes (both performance and learning). As in the previous section, this result could be due to the lack of actions taken by individuals who endorse a fixed mindset when encountering critical feedback that challenges their abilities to design posters (Blackwell et al., 2007). In a mindset intervention, it was found that fixed mindset predicted a flat trajectory in junior-high students’ grades, while a growth mindset predicted an increase in grades (Blackwell et al., 2007). It could also be that the discomfort of interacting with critical feedback persisted across the game for players endorsing more of a fixed mindset and interfered with their focus during the task and, thus, with their capacity to perform well and learn (Lee et al., 2012). It is surprising that individuals endorsing a growth mindset do not reap the benefits of critical feedback to improve their learning outcomes, as related research would suggest in a population of college students (Mangels et al., 2006). For instance, in a recent study exploring the neural underpinnings of mindset, growth mindset was associated with awareness of and attention to mistakes, with individuals who endorsed a growth mindset showing better accuracy after mistakes compared with those who endorsed a fixed mindset (Moser et al., 2011). This study suggested that endorsing a growth mind is characterized by better functionality of a self-monitoring and control system. However, more data are needed to deeply understand the relation between critical feedback and learning outcomes because, in prior research, students who chose higher amounts of critical feedback significantly outperformed those who chose lower amounts of critical feedback but only when they were endorsing higher levels of a growth mindset (Cutumisu, 2019). As this result was not replicated in the Assign condition, perhaps the size of the sample could not detect differences between conditions; future research will explore whether revising mediates the relation between critical feedback and learning outcomes in both conditions.

Students’ personal goals and perceived task difficulty may also influence their motivational orientations and actions. More research is warranted to separate these factors from the influence of mindset on learning behaviors and learning outcomes to gain an insight into the processes and mechanisms that unfold when feedback valence agency, mindset, and learning behaviors interact to impact performance. This is especially important to examine in the presence of more high-stakes, difficult tasks than poster design (e.g., a mathematics domain) and a larger overall sample, in which case students’ cognitive and non-cognitive behaviors may change. For instance, research studies revealed that individuals may behave differently when facing challenges, depending on their mindsets (Dweck, 1999; Blackwell et al., 2007). However, in prior research, middle-school students were able to transfer their critical feedback and revising choices from a classroom environment to the Posterlet game environment, indicating that learning behaviors can transfer, benefitting lower achievers the most (Chin et al., 2019).

#### Feedback Valence Agency, Mindset, Revision, and Learning Outcomes

Results show that feedback valence agency moderates the effect of growth mindset (but not that of fixed mindset) on the relation between the choice to revise and performance but not learning of graphic design principles (i.e., students’ improved perception of the graphic design principles). This result suggests that, although not associated with revision or performance, growth mindset shapes how learners revise their work to improve their poster designs. Thus, the moderating effect of growth mindset on the association between revision and performance differs depending on whether students choose or are assigned their feedback valence. Follow-up analyses showed that the moderation effect of growth mindset on the relation between revising and performance was significant in the Choose but not in the Assign condition.

It is possible that growth mindset is more important for the decision to revise posters and, thus, to improve performance, when students feel that they have autonomy and control over their feedback valence, in line with the principles of self-determination theory. Students in the Choose condition who chose to revise their posters more often (two or three times) rather than less often (up to one time) performed significantly better in the game only when they endorsed a higher rather than a lower level of growth mindset. This result suggests the importance of growth mindset in perhaps sustaining learners’ motivation during the poster design activity, especially when learners are provided with more control over their learning. Growth mindset may drive students to become more intentional while revising their posters and, thus, use this strategy to improve future posters only when they have more feedback agency.

No feedback agency moderation effect of mindset was found for the relation between revising and learning of graphic design principles for both growth and fixed mindset. It seems that feedback valence agency moderates the effect of growth mindset on the relation between revising and performance, but it does not moderate the effect of mindset in general on the relation between learning behaviors and learning of graphic design principles. Also, it is possible that endorsing a fixed mindset influences the way students revise their posters to perform better (which was not found in this experiment) but this does not influence how well they learn. Thus, more research is needed to examine whether students’ goal orientation (performance versus mastery) influences students’ revising behavior from the perspective of performance and learning improvement.

Taken together, results show that although students’ mindset orientation is not related to their revising behavior, mindset seems to determine how students’ revising decisions improve their performance but not learning from the Posterlet task. This is concordant with prior research showing that students endorsing higher levels of growth mindsets tend to focus on the learning process and to construe critical feedback as a learning opportunity rather than a hindrance, thus performing better (Dweck, 1999). Thus, although mindset was also not directly associated with performance, mindset moderated the relation between learning behaviors and learning outcomes. This result was echoed in the related literature indicating that although mindset predicts task motivation, it has a weaker effect on performance, and a potential impact on performance would be indirect, possibly through other factors, such as motivation (Burnette et al., 2013; Sisk et al., 2018). For example, junior-high students who endorsed a growth mindset were able to maintain their motivation in comparison to those endorsing a fixed mindset and, as a result, they increased their grades over the next two years (Blackwell et al., 2007). As motivation has not been directly measured and the size of the dataset does not allow for mediation analyses, in a future study, variables measuring motivational aspects related to poster design will be explored and their direct and indirect effects on mindset, learning behaviors, and learning outcomes will be explored.

Finally, the slight discrepancy in the pattern of results for fixed and growth mindset across choosing or being assigned feedback valence was also echoed in a study that found mindset differences in behavioral responses to critical feedback information (Mangels et al., 2006). This discrepancy also brings more evidence to support the notion that fixed and growth mindsets are distinct mindset constructs and learners usually endorse different degrees of the two mindsets. Although this is still a matter of academic debate, related research supports this conjecture, suggesting that learners endorse a mix of both mindsets (Mercer and Ryan, 2010) and that fixed and growth mindsets are not the same construct (Lou and Noels, 2017; Lou, 2019), representing two dynamic meaning-making systems (Molden and Dweck, 2006).

### Limitations

One of the limitations of this experiment stems from the use of a participant sample of convenience. Thus, results may not generalize to populations different from pre-service teachers who may have a different pattern of mindset compared to the general undergraduate student population. Also, the sample was not large enough for a structural equation modeling analysis, therefore, more data collection is under way. Thus, results may change, especially in the case of the marginally significant results obtained in this study. It is also possible that, because the game may have seemed more geared toward younger participants, some students enjoyed the activity and took the task more seriously than others. Thus, the relations among feedback valence agency, mindset, and learning variables may differ depending on the level of enjoyment or experience with the task domain. However, a recent study has shown that the choice to seek feedback as well as the relation between critical feedback-seeking and learning performance do not vary by age for 727 participants ranging from middle-schoolers to older adults (Cutumisu and Schwartz, 2014). Moreover, this study focuses on participants’ choices and behaviors following their engagement with chosen or assigned critical feedback, rather than on the actual game or poster design activities. Future studies will consider a different task and compare results, including the probing of each task’s suitability to explore the influence of mindset. The poster design task in Posterlet was specifically selected as it constitutes a creative open-ended task, where students usually have comparable levels of experience, in contrast to a more well-structured, procedural scenario (e.g., a mathematics problem), which sets this research apart. Also, Posterlet is an instance of a dynamic assessment (Vygotsky, 1997) in which students have opportunities to learn, thus, the consequences of players’ encounters with critical feedback can be measured as well. The importance of the integration of feedback in an ongoing task has been emphasized in the literature (Black and Wiliam, 2009). Although the game had three rounds, offering players the opportunity to integrate the feedback from one round on a subsequent round, it is possible that students may not have appreciated the value and relevance of feedback to future tasks, which has been found to be an important aspect of engaging more deeply with feedback (Havnes et al., 2012; Evans, 2013; Gamlem and Smith, 2013). Future studies will examine enjoyment between conditions and the relations among enjoyment, mindset, learning behaviors, and learning outcomes. Also, the post-test used in this experiment and detailed in prior work measures a small subset of graphic design principles, which students may not have encountered in the feedback provided by the game, and it has been shown to differentiate between students who played Posterlet and those who did not (Cutumisu et al., 2019). In the future, the post-test will be further refined and used to measure learning of graphic design principles, as it is possible that a better appraisal of students’ learning could yield different results.

Due to constraints regarding the testing sequence, the mindset survey was administered at the end of the Posterlet game, as part of the post-test. Future studies will examine whether the timing of the survey with regard to the Posterlet game impacts the current results, although mindset is believed to be a fairly stable construct. A longitudinal mindset study will be conducted to better understand the potential change in mindset over time.

The differences in behavior observed after seeking or being assigned critical feedback could depend on individual differences, such as mood, anxiety, or university culture. Now that the study has demonstrated that mindset is not related to the decision to seek critical feedback and to revise, regardless of students’ feedback valence agency, future research can focus on discovering why and under what circumstances students seek critical feedback and decide to revise their work.

### Educational Implications

Findings suggest that feedback valence agency plays an important role in how growth mindset and revising influence performance improvement. The unique contribution of the present study is the emphasis on the cumulative weight of feedback valence agency and growth mindset on learning behavior (i.e., revising one’s work) and learning outcomes (i.e., performance) through an experimental yoked study design. Theoretical implications of this research include the clarification of the role of feedback valence agency in shaping the effect of mindset on the relation between learning behaviors and learning outcomes, showing the importance of combining the opportunity to choose one’s feedback valence with the endorsement of a growth mindset to reap the performance benefits of revising one’s work. The experiment suggests that students who are given the chance to choose their feedback valence are more motivated to revise their work and, thus, improve their performance. The moderating effect found in this experiment shows that the lack of associations between mindset and the rest of the variables in both conditions was not due to measurement error, as results show that the role of mindset in shaping the relation between learning behaviors and outcomes is differential, depending on whether students have control or not over their feedback valence. Prior research found that academic success is not only dependent on cognitive ability but also on individuals’ beliefs about their learning (Dweck, 2006). The present study found that the effect of individuals’ beliefs about intelligence on the relation between their revising behavior and their performance is moderated by feedback agency. Practical implications include the design and implementation of assessment environments that include choices and consider psychological attributes such as mindset as a way to improve performance in a digital game. Thus, growth mindset interventions corroborated with design thinking interventions (i.e., revising or creating several drafts and prototypes) to enhance students’ performance would benefit from considering the level of control that students have over the type of feedback they encounter. Such interventions would also serve to protect students from the perceived deleterious effects of critical feedback and to prepare them to cope with failure. Prior research has demonstrated success in improving growth mindset, and in turn achievement (Aronson et al., 2002; Blackwell et al., 2007), as well as in facilitating students’ adoption of a growth mindset by praising their effort instead of ability and encouraging a focus on the learning process rather than on performance comparisons (Yeager and Dweck, 2012; Yeager et al., 2016b). Based on these preliminary results, more research is warranted to gain an insight into the processes and mechanisms that unfold when feedback valence agency, mindset, and critical feedback interact to impact performance.

## Conclusions

This study examined empirically for the first time whether students’ feedback valence agency moderates the effect that mindset (growth and fixed) may have on the relations between learning behaviors (critical feedback and revising) and learning outcomes (poster performance and learning of graphic design principles) in a sample of pre-service teachers. The experiment established the causality of feedback valence agency, showing that feedback valence agency moderated the effect of growth mindset on the relation between revising and performance. Although mindset does not influence students’ decision to revise posters, regardless of their feedback valence agency, growth mindset determines how students make use of their learning choices to improve their performance. This research brings additional empirical evidence suggesting that fixed and growth mindsets measure different mindset constructs, as only growth mindset moderates the relations between revising and performance. Overall, results suggest that growth mindset, feedback-seeking, and revising interventions aimed at improving performance would benefit from considering feedback valence choice, especially as the moderating role of growth mindset in shaping the relation between revising and performance occurred only when students could choose the valence of their feedback.

## Ethics Statement

This study was carried out in accordance with the recommendations of University of Alberta REB committee protocol Pro00059774 with written informed consent from all subjects. All subjects gave written informed consent in accordance with the Declaration of Helsinki. The protocol was approved by the University of Alberta REB committee.

## Author Contributions

The author confirms being the sole contributor of this work and has approved it for publication.

### Conflict of Interest Statement

The author declares that the research was conducted in the absence of any commercial or financial relationships that could be construed as a potential conflict of interest.
